# Impact of long-term exposure to ambient ozone on lung function over a course of 20 years (The ECRHS study): a prospective cohort study in adults

**DOI:** 10.1016/j.lanepe.2023.100729

**Published:** 2023-09-01

**Authors:** Tianyu Zhao, Iana Markevych, Elaine Fuertes, Kees de Hoogh, Simone Accordini, Anne Boudier, Lidia Casas, Bertil Forsberg, Judith Garcia Aymerich, Marco Gnesi, Mathias Holm, Christer Janson, Deborah Jarvis, Ane Johannessen, Rudolf A. Jörres, Stefan Karrasch, Benedicte Leynaert, José Antonio Maldonado Perez, Andrei Malinovschi, Jesús Martínez-Moratalla, Lars Modig, Dennis Nowak, James Potts, Nicole Probst-Hensch, José Luis Sánchez-Ramos, Valerie Siroux, Isabel Urrutia Landa, Danielle Vienneau, Simona Villani, Bénédicte Jacquemin, Joachim Heinrich

**Affiliations:** a Institute and Clinic for Occupational, Social and Environmental Medicine, University Hospital, LMU Munich, Munich, Germany; b Comprehensive Pneumology Center Munich (CPC-M), German Center for Lung Research (DZL), Munich, Germany; c Institute of Epidemiology, Helmholtz Zentrum München - German Research Center for Environmental Health, Neuherberg, Germany; d Institute of Psychology, Jagiellonian University, Krakow, Poland; e “Health and Quality of Life in a Green and Sustainable Environment”, SRIPD, Medical University of Plovdiv, Plovdiv, Bulgaria; f National Heart and Lung Institute, Imperial College London, London, United Kingdom; g MRC Centre for Environment & Health, London, UK; h Swiss Tropical and Public Health Institute, Allschwil, Switzerland; i University of Basel, Basel, Switzerland; j Unit of Epidemiology and Medical Statistics, Department of Diagnostics and Public Health, University of Verona, Verona, Italy; k University Grenoble Alpes, Inserm U 1209, CNRS UMR 5309, Team of Environmental Epidemiology Applied to the Development and Respiratory Health, Institute for Advanced Biosciences, Grenoble, France; l Pediatric Department, CHU Grenoble Alpes, Grenoble, France; m Social Epidemiology and Health Policy, Department of Family Medicine and Population Health, Faculty of Medicine and Health Sciences, University of Antwerp, Antwerp, Belgium; n Institute for Environment and Sustainable Development (IMDO), University of Antwerp, Belgium; o Section of Sustainable Health, Department of Public Health and Clinical Medicine, Umeå University, Umeå, Sweden; p ISGlobal, Barcelona, Spain; q Universitat Pompeu Fabra (UPF), Barcelona, Spain; r CIBER Epidemiología y Salud Pública (CIBERESP), Barcelona, Spain; s Unit of Biostatistics and Clinical Epidemiology, Department of Public Health, Experimental and Forensic Medicine, University of Pavia, Pavia, Italy; t Occupational and Environmental Medicine, School of Public Health and Community Medicine, Institute of Medicine, Sahlgrenska Academy, University of Gothenburg, Gothenburg, Sweden; u Department of Medical Sciences, Respiratory, Allergy and Sleep Research, Uppsala University, Uppsala, Sweden; v Department of Global Public Health and Primary Care, University of Bergen, Bergen, Norway; w Université Paris-Saclay, UVSQ, University Paris-Sud, Inserm, Center for Epidemiology and Population Health (CESP) - Integrative Respiratory Epidemiology Team, 94807, Villejuif, France; x Sección de Neumología, Hospital Juan Ramón Jiménez, Huelva, Spain; y Department of Medical Sciences, Clinical Physiology, Uppsala University, Uppsala, Sweden; z Servicio de Neumología del Complejo Hospitalario Universitario de Albacete, Albacete, Spain; aa Department of Nursing, University of Huelva, Huelva, Spain; ab Department of Pneumology, Galdakao Hospital, Galdakao, Spain; ac University Rennes, Inserm, EHESP, Irset (Institut de recherche en Santé, Environnement et travail), UMR_S 1085, F-35000 Rennes, France; ad School of Public Health and Preventive Medicine, Monash University, Melbourne, Australia

**Keywords:** Air pollution, NDVI, Spirometry, Vital capacity, Forced expiratory volume, Middle aged

## Abstract

**Background:**

While the adverse effects of short-term ambient ozone exposure on lung function are well-documented, the impact of long-term exposure remains poorly understood, especially in adults.

**Methods:**

We aimed to investigate the association between long-term ozone exposure and lung function decline. The 3014 participants were drawn from 17 centers across eight countries, all of which were from the European Community Respiratory Health Survey (ECRHS). Spirometry was conducted to measure pre-bronchodilation forced expiratory volume in 1 s (FEV_1_) and forced vital capacity (FVC) at approximately 35, 44, and 55 years of age. We assigned annual mean values of daily maximum running 8-h average ozone concentrations to individual residential addresses. Adjustments were made for PM_2.5_, NO_2_, and greenness. To capture the ozone-related change in spirometric parameters, our linear mixed effects regression models included an interaction term between long-term ozone exposure and age.

**Findings:**

Mean ambient ozone concentrations were approximately 65 μg/m³. A one interquartile range increase of 7 μg/m³ in ozone was associated with a faster decline in FEV_1_ of −2.08 mL/year (95% confidence interval: −2.79, −1.36) and in FVC of −2.86 mL/year (−3.73, −1.99) mL/year over the study period. Associations were robust after adjusting for PM_2.5_, NO_2_, and greenness. The associations were more pronounced in residents of northern Europe and individuals who were older at baseline. No consistent associations were detected with the FEV_1_/FVC ratio.

**Interpretation:**

Long-term exposure to elevated ambient ozone concentrations was associated with a faster decline of spirometric lung function among middle-aged European adults over a 20-year period.

**Funding:**

10.13039/501100001659German Research Foundation.

**Research in context**

**Evidence before this study**

A comprehensive literature search was conducted on December 16, 2022, to identify relevant studies on the association between ozone exposure and lung function. PubMed, Web of Science, and appropriate governmental websites were searched without restrictions on database inception and language. The search terms used were (“ozone” OR “o3”) AND (“lung function” OR “FVC” OR “FEV1”). The focus was on studies investigating the long-term effects of ozone exposure in adults.

The search yielded a limited number of studies. We also found prior to 2013, the three most relevant studies were included in the United States Environmental Protection Agency's 2020 Integrated Science Assessment (ISA). A systematic review, updated as part of the ISA, covered two studies published between 2013 and 2020. Furthermore, only two of the five studies were longitudinal in nature, and both were conducted in the United States. No studies had examined the long-term change in lung function associated with ambient ozone exposure.

**Added value of this study**

This study provides unique contributions to the existing literature. It is the first longitudinal study on the long-term effects of ozone exposure involving multiple study centers and countries. Our analysis included 3014 European adults from 17 study centers across eight countries. The results consistently demonstrated that higher ambient ozone concentrations were associated with faster declines in spirometric lung function. Notably, the decline pattern observed was indicative of a restrictive rather than an obstructive lung function impairment. These associations remained robust across various models, even after adjusting for co-pollutants and greenness. Additionally, our findings indicated that residents of northern Europe and individuals with higher baseline age exhibited a greater ozone-related decline in lung function.

**Implications of all the available evidence**

This longitudinal study significantly contributes to establishing a potential causal relationship between long-term ambient ozone exposure and lung function decline in adults. Our findings highlight the role of ambient ozone in driving changes in lung function and have important implications for policy development aimed at protecting vulnerable populations. The evidence presented in this study adds to the existing knowledge base and can inform strategies to mitigate the adverse effects of ambient ozone exposure on lung health.

## Introduction

Since the 1960s, research has consistently shown that short-term exposure to ozone is associated with reduced lung function.^1^ In recent years, *in vitro* and animal studies,^2^^,^^3^ as well as many human exposure experiments,^4^ have further substantiated this relationship. The United States Environmental Protection Agency's 2020 Integrated Science Assessment (ISA)^5^ synthesized the available evidence and confirmed the detrimental impact of ozone on lung function in various exposure scenarios. However, the ISA identified only a limited number of studies and reported inconsistent findings regarding the association between long-term ozone exposure and lung function.^5^

To address this knowledge gap, an updated systematic review from the ISA was published in 2022.^6^ This review focused on epidemiological studies conducted between 2013 and 2020 and found evidence of an association between short-term ozone exposure and lung function in children. Additionally, long-term ozone exposure was linked to lung function levels and growth in children. Although a similar association was observed in adults for short-term exposure, the evidence for long-term exposure was limited to just two new studies^7^^,^^8^ from the United States, rendering it inconclusive.

Given the scarcity of epidemiological studies investigating the relationship between long-term ozone exposure and changes in lung function among adults, a comprehensive study on this topic is warranted. It is worth noting that the relationship between ozone and vegetation may also play a role.^9^ Local vegetation levels have been correlated with ozone concentrations, and the association between living in a green neighborhood and lung function has yielded inconsistent findings.^10^, ^11^, ^12^ Therefore, it would be advantageous for a study on ozone exposure and lung function to consider the additional influence of greenspace.

In this study, we aimed to explore the association between ambient ozone exposure and spirometric lung function in adults over a 20-year period using a multicenter cohort. Furthermore, we sought to examine whether co-exposure to particulate matter with an aerodynamic diameter <2.5 μm (PM_2.5_) and nitrogen dioxide (NO_2_) at residential locations and residing in greener areas could confound the observed association.

## Methods

### Study population and design

The European Community Respiratory Health Survey (ECRHS; www.ecrhs.org) is an international prospective multicenter cohort study aimed at investigating respiratory health. The study initially recruited over 18,000 participants aged 20–44 years from 30 centers in 14 countries between 1990 and 1994. The recruitment process involved a random sample from population-based registers and an oversampling of participants with respiratory symptoms.^13^

The baseline enrollment, known as ECRHS I, consisted of a questionnaire survey and a medical examination.^13^ Subsequent follow-up surveys were conducted during ECRHS II (1999–2003) when participants were between the ages of 27 and 57, and ECRHS III (2010–2014) when participants were between the ages of 39 and 67.^14^ Ethical approval was obtained from the relevant ethics committees in each center, complying with national legislation, and all participants provided written informed consent.

**Fig. 1:**
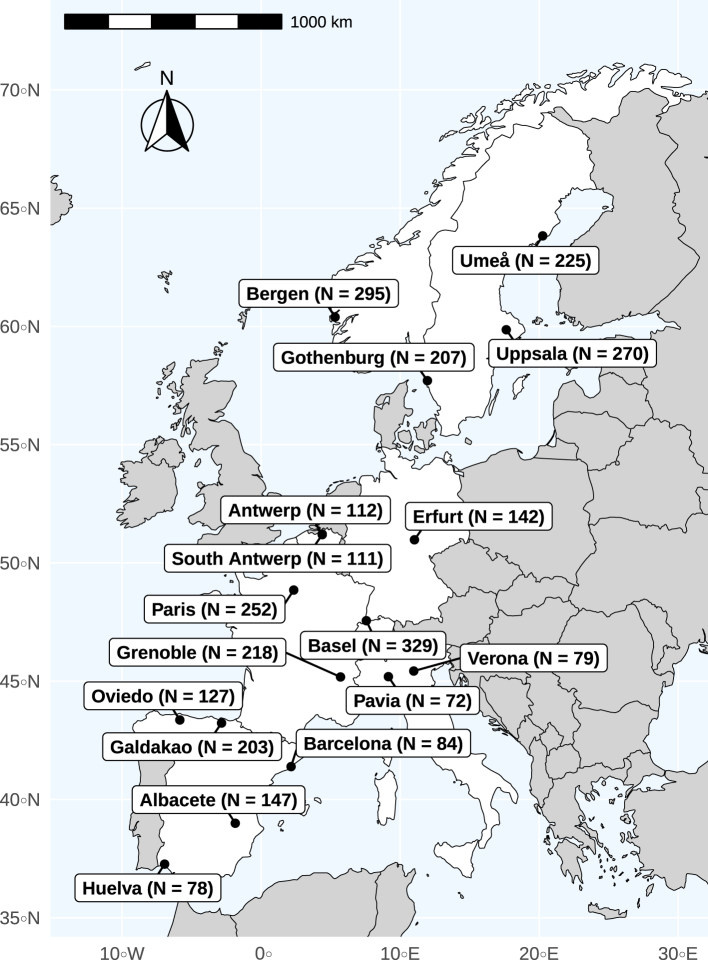
Included ECRHS study centers and numbers of available participants in each center.

To explore the determinants of lung function decline, this longitudinal study focused on participants older than 25 years^12^^,^^15^ at ECRHS I who had at least two lung function measurements. The study population was further refined based on the availability of ozone data, resulting in a final sample of 3014 participants from 17 centers across eight countries. The selected centers included Northern Europe—Umeå, Uppsala, and Gothenburg in Sweden, Bergen in Norway; Central Europe—Antwerp city and South Antwerp in Belgium, Erfurt in Germany, Paris and Grenoble in France, Basel in Switzerland, Verona and Pavia in Italy; and Southern Europe—Oviedo, Galdakao, Barcelona, Albacete, and Huelva in Spain. The study population selection process is illustrated in a flowchart (Appendix p 3), and the geographic distribution of the study centers is depicted in Fig. 1.

### Lung function

During each study visit, participants underwent standardized spirometric measurements following the recommendations of the American Thoracic Society and European Respiratory Society.^16^, ^17^, ^18^ The spirometric parameters assessed were forced expiratory volume in 1 s (FEV_1_) and forced vital capacity (FVC), measured without prior bronchodilation. To ensure consistency and minimize measurement biases, calibration, which simultaneously considered age, self-reported sex (hereafter referred to as sex), and height, had been performed to account for variations in the spirometers used across different centers and follow-up visits (Appendix p 4).^19^

FEV_1_, FVC, and the FEV_1_/FVC ratio were the outcomes of this analysis. FEV_1_ is the volume of air that can be exhaled during the first second of forced exhalation after full inspiration, while FVC is the total amount of air exhalation during the test. The FEV_1_/FVC ratio can be used to distinguish between obstructive and restrictive lung disorders.^20^ An obstructive pattern is characterized by a reduced FEV_1_/FVC ratio, indicating airflow limitation, whereas a restrictive pattern is characterized by reduced FVC relative to predicted values, suggesting a decrease in lung volume.

### Assessment of ambient air pollution exposure

Annual mean concentrations (μg/m³) of ozone, PM_2.5_, and NO_2_ modeled to the geocoded residential addresses of participants during each ECRHS survey were utilized as proxies of long-term air pollution, in line with our previous study.^21^ Concentrations of all pollutants were obtained from the Effects of Low-Level Air Pollution: A Study in Europe project (ELAPSE; www.elapseproject.eu).^22^ Annual mean concentrations of ozone were calculated as the averages of daily maximum running 8-h average concentrations, while PM_2.5_ and NO_2_ levels were estimated as annual means. In brief, ELAPSE developed land-use regression models on a West–European scale, utilizing a spatial resolution of 100 m × 100 m. The models were based on the AirBase v8 dataset (www.eea.europa.eu/data-and-maps/data/aqereporting-9) and incorporated additional data from satellite observations, chemical transport models, land use information, and traffic predictors. To account for residual spatial autocorrelation, kriging on residuals was also applied in the ELAPSE models for pollutants such as ozone, which exhibit strong regional variations. Explained spatial variations (R squared) of the models were approximately 65% for ozone, 72% for PM_2.5_, and 59% for NO_2_.

While ELAPSE initially focused on the year 2010, back- and forward-extrapolation methods utilizing a chemical transport model were employed to estimate annual concentrations for other years.^23^ This allowed for the derivation of time-varying long-term exposures to ozone, PM_2.5_, and NO_2_ at the geocoded residential addresses of participants during each survey round of the ECRHS. The assignment of these exposure values was conducted using ArcGIS software (version 10.4, ESRI, Redlands, CA).

### Residential greenspace

Individual exposure to greenspace was assessed using satellite-based greenness measurements. Consistent with our previous study on greenspace exposure,^12^ we utilized the normalized difference vegetation index (NDVI)^24^ as the metric to capture the surrounding greenness or vegetation density. The NDVI, calculated from spectral reflectance measurements obtained in specific wavelengths relevant to vegetation, produces values ranging from −1 to +1. Higher NDVI values indicate a greater degree of vegetation presence.

Briefly, to derive NDVI values, we utilized Landsat 4–5 Thematic Mapper satellite images (http://earthexplorer.usgs.gov). Cloud-free satellite images with a spatial resolution of 30 m × 30 m were selected specifically during the months when vegetation is most abundant. We then calculated residential greenness by averaging the NDVI values within a circular buffer of a 300 m radius around each participant's residential address during each round of the ECRHS.

### Covariates

We collected information on participants’ characteristics, including type of sample, sex, age, height, weight, body mass index (BMI), education, occupation, smoking status, and urbanicity. A detailed description of covariates is presented in the Supplementary (Appendix p 5). In addition, the annual average temperature of each center was collected from national or local meteorological institutes.

### Statistical analysis

#### Main analysis

We performed Chi-square tests and Student's t-tests to identify differences between the analytical sample and that initially recruited in the ECRHS (aged >25 years). In order to examine potential non-linear relationships, we initially explored deviations from linearity by categorizing the ozone data into tertiles and quartiles. However, no indications of non-linearity were observed in these analyses (data not shown). Hence, to assess the associations between annual ozone exposure and changes in FVC, FEV_1_, and the FEV_1_/FVC ratio, we employed multivariable mixed linear regression models with random intercepts. These models were fitted using the *lmer* function from the *lme4* package^25^ in the statistical program R, version 4.1.2.^26^ Within the model, the inclusion of a non-equal number of measurements for different participants can be balanced. With the longitudinal data, effect estimates are less prone to regional confounding and subtle selection factors as each subject serves as its own control. We included an interaction term between ozone and age at lung function measurement which allowed us to capture the ozone-related changes in the lung function parameter over time. Consequently, the resulting effect estimate can be expressed in mL/year for FEV_1_ and FVC, and %/year for the FEV_1_/FVC ratio.

The selection of covariates for the statistical models was supported by a directed acyclic graph (DAG, Appendix p 6) and guided by evidence and knowledge from previous studies.^12^^,^^27^ The main covariates included in the models were type of sample, sex, age (including an age-squared term), height, weight, education, occupation, smoking status, and lifetime pack-years. We centered continuous variables over the data.

#### Sensitivity analyses

Several additional analyses were performed to confirm the robustness of the association between ambient ozone exposure and lung function parameters. First, minimally adjusted models were built, including only the type of sample, sex, age, age squared, and height. Second, the main models were further adjusted for (1) season, (2) co-pollutants (negatively correlated with ozone, correlation of PM_2.5_ was weak while of NO_2_ was moderate; Appendix p 8), (3) NDVI (weakly correlated with ozone; Appendix p 8), (4) spirometer model (Appendix p 4), (5) spirometric indices at the baseline survey, (6) urbanicity, (7) temperature, and (8) continuous or categorized BMI instead of height and weight. Additionally, the analytic population of the main analysis was restricted to (1) participants who had lung function and ozone data at all three ECRHS surveys, (2) a sample without the Spanish centers, for which addresses at ECRHS I could not be retrieved, (3) participants who never changed their place of residence, (4) non-smokers, (5) the random sample of participants from population-based registers, (6) those who never reported an asthma attack in the last 12 months and are not currently taking asthma medication and (7) participants who never reported hay fever or other allergies with similar symptoms. A model including centers as a fixed effect rather than a random effect was built as well.

#### Stratified analyses

We conducted several stratified analyses to test for potential effect modification by (1) sex, (2) median age at ECRHS I (≤35 years, >35 years), (3) age of completion of full-time education (<17 years, 17–20 years, and >20 years), (4) occupation (non-manual, manual), (5) urbanicity at ECRHS I (urban, suburban/rural, as there were too few suburban residents to analyze separately), and (6) geographic region (Northern Europe, Central Europe, and Southern Europe).

### Role of the funding source

The funding sources of this study had no role in the study design, the collection, analysis, and interpretation of data, the writing of the report, and in the decision to submit the article for publication.

## Results

### Characteristics of study population

Characteristics of the included 3014 participants are listed in Table 1. About half of the 3014 ECRHS participants included in this analysis were females (51.3%). Nearly half were central European residents (45.7%), while one-third (33.1%) and less than a quarter (21.2%) were from Northern Europe and Southern Europe, respectively (Table 1).

**Table 1:** Characteristics of study population.

Characteristic	ECRHS I		ECRHS II		ECRHS III	
	n/N or N	% or mean ± SD	n/N or N	% or mean ± SD	n/N or N	% or mean ± SD
Sex						
Female	1545/3014	51.3				
Male	1469/3014	48.7				
Age, years	3014	35.4 ± 6.1	3012	44.3 ± 6.1	3014	55.3 ± 6.1
Height, cm	2864	170.2 ± 9.7	2866	170.0 ± 9.8	2822	169.4 ± 9.8
Weight, kg	2864	69.8 ± 13.5	2855	74.1 ± 15.0	2800	77.5 ± 15.8
BMI, kg/m^2^	2864	24.0 ± 3.8	2855	25.6 ± 4.3	2800	26.9 ± 4.7
Geographic area^a^						
Northern Europe	997/3014	33.1				
Central Europe	1378/3014	45.7				
Southern Europe	639/3014	21.2				
Age at completion of full-time education						
<17 years			472/3008	15.7		
17–20 years			974/3008	32.4		
>20 years			1562/3008	51.9		
Occupation						
Management/professional/non-manual					693/3014	23.0
Technical/professional/non-manual					568/3014	18.8
Other non-manual					789/3014	26.2
Skilled manual					267/3014	8.9
Semi-skilled/unskilled manual					400/3014	13.3
Other/unknown					297/3014	9.9
Smoking						
Never	1271/2978	42.7	1216/2748	44.3	1153/2499	46.1
Ex-smoker with <15 pack-years	533/2978	17.9	534/2748	19.4	489/2499	19.6
Ex-smoker with ≥15 pack-years	179/2978	6.0	305/2748	11.1	417/2499	16.7
Current smoker with <15 pack-years	570/2978	19.1	232/2748	8.4	92/2499	3.7
Current smoker with ≥15 pack-years	425/2978	14.3	461/2748	16.8	348/2499	13.9
Urbanicity^b^						
Urban	1797/2354	76.3	2110/3009	70.1	2024/3004	67.4
Semi-urban	54/2354	2.3	334/3009	11.1	387/3004	12.9
Rural	503/2354	21.4	565/3009	18.8	593/3004	19.7
Lung function						
FEV_1_, mL	2189	3759.0.±817.0	2827	3456.0 ± 796.1	2749	2980.8 ± 748.0
FVC, mL	2147	4606.9 ± 1031.9	2800	4337.1 ± 991.1	2714	3943.4 ± 951.6
FEV_1_/FVC, %	2119	81.8 ± 7.0	2790	79.9 ± 6.6	2701	75.6 ± 6.7

Abbreviations: BMI, body-mass-index; ECRHS, European Community Respiratory Health Survey; FEV_1_, forced expiratory volume in 1 s; FVC, forced vital capacity; SD, standard deviation.

a Umeå, Uppsala, Gothenburg, and Bergen as Northern Europe; Antwerp city, South Antwerp, Erfurt, Paris, Grenoble, Basel, Verona, and Pavia as Central Europe; and Oviedo, Galdakao, Barcelona, Albacete, and Huelva as Southern Europe.

b According to the standard EU Degree of Urbanisation1 classification for the year 2001, urban/cities: densely populated areas, semi-urban/towns and suburbs: intermediate density areas, and rural areas: thinly populated areas.

The participants’ mean ages were 35, 44, and 55 years at the three follow-ups, and mean FEV_1_ was 3759, 3456, and 2980 mL at ECRHS I, II, and III, respectively, showing the expected decrease of absolute values with increasing age. Likewise, the mean FVC was 4606, 4337, and 3943 mL, respectively.

The analytical sample did not statistically differ from those originally recruited in the ECRHS (>25 years), except that the selected participants had higher education and occupation levels, as well as higher FEV_1_ and FVC values at ECRHS I.

### Characteristics of geographical variables

**Fig. 2:**
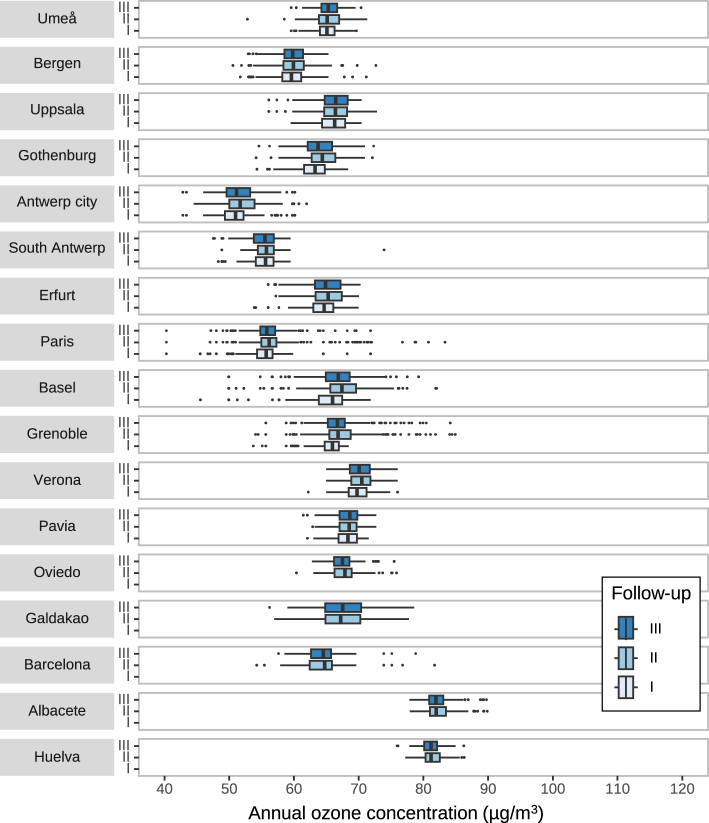
Distribution of annual ozone concentrations across three ECRHS surveys and 17 centers.

Median ambient ozone concentrations were 66 μg/m³ at ECRHS II and III and were higher compared to 64 μg/m³ at ECRHS I (Table 2 and Fig. 2). Across all centers, higher levels of ozone were observed in southern European centers, especially Albacete and Huelva, while Antwerp and Paris had the lowest levels (Fig. 2). Median concentrations of PM_2.5_ and NO_2_ were around 27 μg/m³ and 16 μg/m³, respectively (Table 2). Levels of ozone, PM_2.5_, and NO_2_ varied across the study centers, but the changes in their concentrations across the three surveys were small (Table 2 and Appendix p 9–10), and NDVI levels had similar patterns (Table 2 and Appendix p 11). The southern European Spanish centers had lower levels, while the Swedish and Norwegian centers had higher NDVI levels (Appendix p 11).

**Table 2:** Distribution of ambient ozone and other exposure.

Exposure	Index	ECRHS I		ECRHS II		ECRHS III	
		n	Concentration	n	Concentration	n	Concentration
Ozone, μg/m³	Median (25th–75th percentile)	2119	63.9 (58.7–66.4)	2790	65.8 (61.3–68.8)	2701	65.5 (61.2–68.3)
PM_2.5_, μg/m³	Median (25th–75th percentile)	2259	17.4 (8.4–19.1)	3014	15.1 (9.0–18.4)	3014	15.2 (9.1–18.4)
NO_2_, μg/m³	Median (25th–75th percentile)	2259	28.1 (19.3–35.2)	3014	25.8 (18.5–32.7)	3014	26.8 (19.0–33.2)
NDVI in 300 m buffer	Mean ± SD	2124	0.28 ± 0.16	2790	0.24 ± 0.17	2701	0.26 ± 0.16

Abbreviations: NDVI, Normalized Difference Vegetation Index; NO_2_, nitrogen dioxide; PM_2.5_, particulate matter with aerodynamic diameter <2.5 μm; SD, standard deviation.

### Relationship between ozone exposure and lung function change

A higher level of ambient ozone was associated with a faster decline in lung function (Table 3 and Appendix p 12). Specifically, an increase of 7 μg/m³ (interquartile range) in the annual average of daily maximum running 8-h average concentrations of ozone was associated with a faster decline in FEV_1_ of −2.08 mL/year (95% confidence interval [CI]: −2.79, −1.36) and FVC of −2.86 mL/year (95% CI, −3.73, −1.99). These associations were robust to adjustments for co-pollutants of PM_2.5_ and NO_2_, and models additionally considering NDVI did not attenuate the effect estimates of ozone. The results regarding the FEV_1_/FVC ratio were largely inconsistent.

**Table 3:** Associations between 7 μg/m³ increase in ambient ozone and lung function change.

Model^a^	FEV_1_ (mL/year)			FVC (mL/year)			FEV_1_/FVC (%/year)		
	n	beta	95% CI	n	beta	95% CI	n	beta	95% CI
Main^b^	**2909**	**−2.08**	**(−2.79, −1.36)**	**2909**	**−2.86**	**(−3.73, −1.99)**	2909	0.005	(−0.007, 0.017)
Minimally adjusted^c^	**3014**	**−1.74**	**(−2.43, −1.04)**	**3014**	**−2.72**	**(−3.57, −1.88)**	3014	0.010	(−0.001, 0.022)
Main + PM_2.5_	**2909**	**−1.73**	**(−2.46, −1.00)**	**2909**	**−2.58**	**(−3.47, −1.69)**	2909	0.008	(−0.004, 0.021)
Main + NO_2_	**2909**	**−1.50**	**(−2.35, −0.65)**	**2909**	**−2.43**	**(−3.46, −1.40)**	2929	0.008	(−0.006, 0.023)
Main + PM_2.5_ + NO_2_	**2929**	**−2.31**	**(−3.26, −1.35)**	**2929**	**−3.20**	**(−4.36, −2.04)**	2909	−0.0002	(−0.017, 0.016)
Main + NDVI	**2909**	**−2.07**	**(−2.79, −1.36)**	**2909**	**−2.86**	**(−3.73, −1.99)**	2909	0.005	(−0.006, 0.018)
Main + PM_2.5_ + NO_2_ + NDVI	**2909**	**−2.05**	**(−2.76, −1.33)**	**2909**	**−2.85**	**(−3.72, −1.98)**	2909	0.006	(−0.006, 0.018)
Main + Spirometer	**2909**	**−1.86**	**(−2.59, −1.12)**	**2909**	**−3.12**	**(−4.02, −2.22)**	**2929**	**0.016**	**(0.004, 0.029)**
Main + Baseline spirometric indices^d^	**2707**	**−1.39**	**(−2.04, −0.74)**	**2678**	**−2.22**	**(−2.96, −1.48)**	**2651**	**0.011**	**(0.004, 0.022)**
Main + Urbanicity	**2906**	**−2.05**	**(−2.77, −1.34)**	**2906**	**−2.83**	**(−3.70, −1.96)**	2906	0.005	(−0.007, 0.409)
Main + Temperature	**2909**	**−2.07**	**(−2.79, −1.36)**	**2909**	**−2.87**	**(−3.74, −2.00)**	2909	0.005	(−0.006, 0.017)
**Selected population**									
Participants in all three surveys	**1559**	**−1.86**	**(−2.84, −0.89)**	**1559**	**−3.81**	**(−4.98, −2.63)**	**1599**	**0.015**	**(0.015, 0.048)**
Never movers	**796**	**−1.93**	**(−3.37, −0.29)**	**796**	**−3.50**	**(−5.51, −1.80)**	**796**	**0.030**	**(0.004, 0.056)**
Non-smokers	**1276**	**−2.42**	**(−3.51, −1.33)**	**1276**	**−2.95**	**(−4.27, −1.64)**	1276	0.001	(−0.017, 0.021)
Participants without asthma	**2754**	**−2.01**	**(−2.73, −1.30)**	**2754**	**−2.54**	**(−3.41, −1.66)**	2754	0.004	(−0.009, 0.016)
Participants without allergic rhinitis	**2305**	**−2.19**	**(−3.05, −1.33)**	**2305**	**−2.89**	**(−3.94, −1.83)**	2305	0.003	(−0.011, 0.018)

Abbreviations: CI, confidence interval; FEV_1_, forced expiratory volume in 1 s; FVC, forced vital capacity; NDVI, Normalized Difference Vegetation Index; NO_2_, nitrogen dioxide; PM_2.5_, particulate matter with aerodynamic diameter <2.5 μm.

Boldface indicates p value < 0.05.

a Sample size of different models changes due to data availability; associations were assess by mixed linear models with an interaction term between long-term ozone exposure and age.

b With random intercepts for subjects nested within centers, and adjusted for sample, sex, age, age squared, height, weight, age at completion of full-time education, occupation, and smoking status and lifetime pack-years smoked.

c With random intercepts for subjects nested within centers, and adjusted for sample, sex, age, age squared, and height.

d With random intercepts for subjects nested within centers, and adjusted for sample, sex, age, age squared, height, weight, age at completion of full-time education, occupation, and smoking status and lifetime pack-years smoked; baseline FEV_1_, FVC, and the FEV_1_/FVC ratio was adjusted in the analysis, respectively.

The observed associations were also consistent in other sensitivity analyses. Adjusting for the spirometer model and further inclusion of baseline spirometric indices, urbanicity, or temperature did not substantially affect the results. The models with fixed effects of centers, alternative adjustment of BMI, or controlling for the season of lung function measurement showed similar associations. The analyses based on specific populations, e.g., non-smokers or participants without asthma or allergic rhinitis, did not materially change the associations.

Overall, the ozone-related faster decline in FEV_1_ and FVC but not the FEV_1_/FVC ratio may indicate a primary long-term effect on lung volume and less on airflow obstruction.

### Stratified analyses

**Fig. 3:**
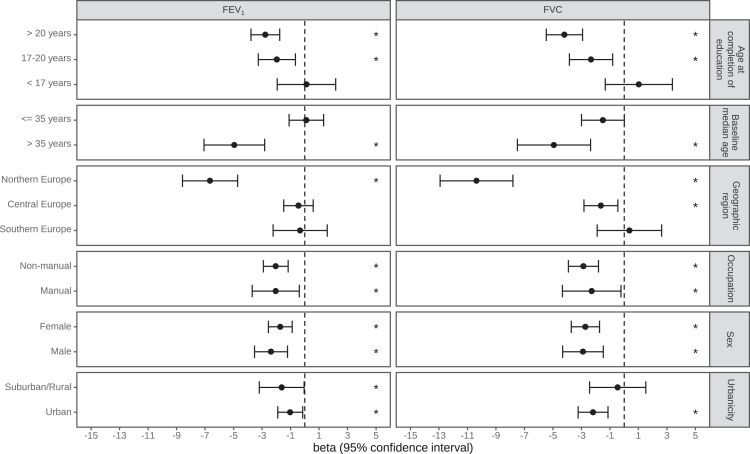
Associations between 7 μg/m³ increase in ambient ozone and FEV_1_ and FVC change (mL/year) over a 20-year period.

The results of the stratified analyses are shown in Fig. 3. There were hints towards potentially stronger associations between higher levels of ozone and faster decline in FEV_1_ and FVC among northern European participants and participants aged above 35 years at baseline. No difference by sex, education, occupation, or urbancity was suggested.

## Discussion

### Main study findings

Based on a comprehensive analysis of 3014 European adults from 17 study centers across eight countries, our study provides compelling evidence linking higher ambient ozone concentrations to faster declines in spirometric indices. This association remained robust across various models, even after adjusting for co-pollutants and NDVI. Notably, FEV_1_ and FVC exhibited similar decline rates, indicating a restrictive pattern of lung function decline rather than an obstructive pattern. However, no consistent associations were observed with the FEV_1_/FVC ratio. Furthermore, our findings revealed that individuals residing in northern Europe and those with higher age experienced a more pronounced decline in lung function in relation to ozone exposure. Although the annual decline in absolute terms may appear modest, the cumulative effect over a 20-year period could lead to a reduction in both FEV_1_ and FVC by approximately 30–40 mL. This reduction corresponds to a spirometric lung function impairment of up to 10 percent compared to the age-related decline.

### Interpretations and comparisons with other studies

Our results are compatible with the findings of the few existing studies. In the U.S., a multicenter cohort of 1874 heavy smokers with or at risk for chronic obstructive pulmonary disease demonstrated that 10-year ozone exposure was associated with reduced postbronchodilator FEV_1_ in terms of %predicted.^7^ Similar results were observed in a second multicenter longitudinal cohort study,^8^ comprising 3636 participants and indicating that a 3-ppb (around 6 μg/m³) increase in long-term ozone levels assessed over ten years was significantly associated with an 18.15 mL greater decline in FEV_1_ (95% CI, 1.59–34.71) and a 40.19 mL greater decline in FVC (95% CI, 17.88–62.49). The associations were not affected by additional adjustments for other ambient air pollutants. Besides the findings from the U.S.,^7^^,^^8^ one recent large, cross-sectional study with 50,991 participants from China also reported associations of long-term ozone exposure with impaired small airway function and higher small airway dysfunction risks.^28^ Interestingly, in the two-pollutant model with adjustment for annual PM_2.5_, which was positively correlated with ozone in the study (Pearson correlation coefficients ranged from 0.4 to 0.7), Niu and colleagues, however, observed a higher FVC with increasing ozone concentrations.^28^

Nordeide Kuiper et al. conducted a cross-sectional study of 3428 Scandinavian adults, including a retrospective assessment of life-long exposures to ozone, other air pollutants, and NDVI.^29^ After categorizing lung function into values either below or above the lower limit of normal (defined as z-score <1.64 standard deviation), the authors reported that childhood and adolescent exposures to ozone were associated with lower values of FEV_1_ and FVC in adulthood. Additional adjustments for NDVI and NO_2_ did not substantially affect the results. Similarly, our model accounting for NDVI did not significantly confound or modify the association between ozone and lung function decline. However, the mechanisms of exposure to vegetation and co-factors are not sufficiently understood,^30^ and the correlation between ozone and urban vegetation is complicated.^9^ Since NDVI cannot provide detailed information on vegetation types, our study hardly uncovered the roles of vegetation on ozone levels. Further comprehensive research is warranted to explore the relationships between ozone, greenspace, and lung function.

The available epidemiological studies differ in terms of study design, population characteristics, lung function characteristics, adjustment factors, and modeling, which makes it difficult to compare findings across studies. It is specifically challenging to compare absolute values of the regression coefficients, as the aforementioned previous studies have expressed their coefficients in mL whereas in the current analysis, results are expressed in mL/year to reflect ozone-related excess decline in lung function over 20 years. Despite the differences, our findings mirrored the results of the most recent European study.^29^ In addition, a comparison with the only recent available longitudinal study based on the general population from the U.S.^8^ seems justified, showing that the results of this study and ours were qualitatively consistent and even quantitatively similar. This was true regardless of the differences in ozone concentrations, with ambient ozone concentrations around 43 μg/m³ in the U.S. study and around 63 μg/m³ in the present study. The effect estimates were of the same order of magnitude for FEV_1_ (−1.85 mL per year versus −2.08 mL per year) and FVC (−4.02 mL per year versus −2.86 mL per year), although the CIs of effect estimates in our study were much smaller compared to the U.S. study.^8^ The differences may be related to another interesting finding of our study. Long-term exposure to ozone appeared to have stronger effects on Nordic countries in our analysis, which have lower ozone levels. It is consistent with the results of the U.S. study^8^ that had similarly low concentrations of ozone. The U.S. study^8^ also reported no significant associations between ozone and the FEV_1_/FVC ratio, in line with our results. As the size of the ozone-related changes in FEV_1_ and FVC are similar and in the same direction, this may mathematically explain why the FEV_1_/FVC ratio appears unaffected.

There are several potential explanations that at present remain speculations, but the most interesting one may be that the low-level average over the whole year for Northern Europe was linked to relatively higher increases in the spring compared to the winter,^31^ and this prevented the persistent activation of mechanisms reducing the effects of ozone on lung function. Southern Europe, on the other hand, has higher ozone concentrations all year round. Remarkably, until now, the experimental findings on repeated ozone exposures, including so-called tolerance regarding lung function responses^32^ versus the different patterns regarding mucosal inflammation^32^ and allergen responsiveness,^33^ have never been addressed in epidemiological studies. It is possible that the long-term effects on lung function are indicative of inflammatory or remodeling impacts on lung function,^34^ while its acute effects reflect neural activation.^35^

Our study also found that individuals above 35 years at baseline exhibited stronger associations between ambient ozone levels and a decline in lung function. This observation suggests that older individuals may be more vulnerable to the adverse effects of ozone exposure on respiratory health.^36^ Given the widespread presence of ambient ozone, the cumulative impact on lung function could contribute significantly to the overall disease burden, particularly among the elderly population.

Furthermore, it is noteworthy that while concentrations of PM_2.5_ and NO_2_ showed a decline over the 20-year study period, ozone levels did not exhibit a similar trend.^37^^,^^38^ Considering the projected impacts of climate change, it is unlikely that ozone levels will decrease in the future.^37^ This implies that the adverse effects of ozone on lung function may persist or even worsen over time. The findings in our study, which require replication in other studies and settings, nonetheless underscore the importance of implementing effective strategies to mitigate ozone pollution^39^^,^^40^ and protect respiratory health, especially among older individuals. It is also crucial to prioritize preventive measures and public health interventions to reduce the burden of ozone-related lung function decline, considering the persistent nature of ozone pollution and its potential to exacerbate respiratory conditions.

### Strengths and limitations

The current work adds substantially to existing knowledge as it is the first longitudinal study on the long-term effects of ozone exposure on lung function decline. This analysis capitalized on the available repeated population-based data collected from multiple centers within several European countries using a standardized protocol and a strong focus on respiratory health. Our results were robust to co-adjustments for spatially correlated co-pollutants and vegetation degree, adjustments for several potential confounding factors, and investigations of effect modification by important covariates, providing confidence in the validity of our findings. Consequently, our results may exhibit a high degree of generalizability.

One of the limitations of this work was that the ECRHS was not specifically designed to examine environmental exposures. Hence, we lack information on certain factors which may have reduced exposure misclassification, such as the mobility pattern of participants (including around their home and at their workplace) and how much time participants spend indoors. Furthermore, we lack a complete record of their residential history. As we had access to the participants’ addresses at the time of the three surveys only, we had to assign the modeled ozone exposure data to these addresses. However, the above measurement bias should be nondifferential, which should cause the observed associations to be attenuated compared to the true associations. It should also be noted that different spirometers were used across the three measurement campaigns for all centers except Verona. Despite lung function measurements being calibrated to reduce measurement biases, the data may still contain a certain degree of noise which may reduce the precision of effect estimates. Although we adjusted our findings for the most important criteria air pollutants, such as PM_2.5_ and NO_2_, how other air pollutants, such as ultrafine particles and black carbon, as well as pollen-derived airborne allergens, might interact to influence the association between ozone exposure and lung function decline remains unknown. As in all large population-based cohorts which have collected data over many decades, there is a risk that some participants may be loss-to-follow, which could introduce selection bias in our results. However, it is worth noting that a prior study using ECRHS data highlighted that estimated exposure-outcome associations might be less influenced by this issue than, for example, prevalence estimate calculations.^41^ Additionally, even though our results remained similar after excluding participants with asthma or allergic rhinitis, we acknowledge that other health conditions not analyzed in this study (e.g., cancers) may have an impact on the investigated associations.

### Conclusion

Our study of middle-aged European adults revealed a significant association between higher long-term ambient ozone levels and a faster decline in spirometric lung function. The decline in FEV_1_ and FVC was similar and thus showed the pattern of a restrictive and not obstructive disorder. This association was independent of concurrent exposure to PM_2.5_ and NO_2_, and it was not mitigated by residing in greener neighborhoods.

Despite the modest yearly decline observed, the cumulative effect over time can lead to a substantial reduction in lung function. This highlights the potential long-term impact of ambient ozone exposure on respiratory health. Future research should further investigate the underlying mechanisms and identify targeted interventions to mitigate the adverse effects of long-term ozone exposure on lung function.

## Contributors

**Tianyu Zhao:** Conceptualization, Data curation, Formal analysis, Methodology, Software, Visualization, Writing–original draft, Writing–review & editing. **Iana Markevych:** Conceptualization, Data curation, Software, Writing–review & editing. **Elaine Fuertes:** Methodology, Writing–review & editing. **Kees de Hoogh:** Data curation, Software, Writing–review & editing. **Simone Accordini:** Writing–review & editing. **Anne Boudier:** Writing–review & editing. **Lidia Casas:** Writing–review & editing. **Bertil Forsberg:** Writing–review & editing. **Judith Garcia Aymerich:** Writing–review & editing. **Marco Gnesi:** Writing–review & editing. **Mathias Holm:** Writing–review & editing. **Christer Janson:** Writing–review & editing. **Deborah Jarvis:** Funding acquisition, Writing–review & editing. **Ane Johannessen:** Writing–review & editing. **Rudolf A. Jörres:** Writing–original draft, Writing–review & editing. **Stefan Karrasch:** Funding acquisition, Writing–review & editing. **Benedicte Leynaert:** Writing–review & editing. **José Antonio Maldonado Perez:** Writing–review & editing. **Andrei Malinovschi:** Writing–review & editing. **Jesús Martínez Moratalla:** Writing–review & editing. **Lars Modig:** Writing–review & editing. **Dennis Nowak:** Funding acquisition, Writing–review & editing**. James Potts:** Data curation, Writing–review & editing. **Nicole Probst-Hensch:** Writing–review & editing. **José Luis Sánchez-Ramos:** Writing–review & editing. **Valerie Siroux:** Writing–review & editing. **Isabel Urrutia Landa:** Writing–review & editing. **Danielle Vienneau:** Writing–review & editing. **Simona Villani:** Writing–review & editing. **Benedicte Jacquemin:** Writing–review & editing. **Joachim Heinrich:** Conceptualization, Funding acquisition, Methodology, Supervision, Writing–original draft, Writing–review & editing.

## Data sharing statement

The ECRHS datasets are not publicly available. The data that support the findings of this study are available from the corresponding author upon reasonable request.

## Editor note

The Lancet Group takes a neutral position with respect to territorial claims in published maps and institutional affiliations.

## Declaration of interests

Marco Gnesi is currently an employee at AstraZeneca SpA; however, the research presented here has been conducted previously and AstraZeneca had no role in any phase of the research project.

The authors have no known competing financial interests or personal relationships that could have appeared to influence the work reported in this paper.
