# Supplementary material for: Impact of long-term exposure to ambient ozone on lung function over a course of 20 years (The ECRHS study): a prospective cohort study in adults
Source: Lancet Reg Health Eur. 2023 Sep 1;34:100729. doi: 10.1016/j.lanepe.2023.100729 (PMC10482740; doi:10.1016/j.lanepe.2023.100729)
Supplement: Supplementary Material [file mmc1.pdf]

Supplementary material

Impact of long-term exposure to ambient ozone on lung function over a course of 20 years (The ECRHS study): a prospective cohort study in adults

Tianyu Zhao[1, 2, 3], Iana Markevych[4, 5], Elaine Fuertes[6, 7], Kees de Hoogh[8, 9], Simone Accordini[10], Anne Boudier[11, 12], Lidia Casas[13, 14], Bertil Forsberg[15], Judith Garcia Aymerich[16, 17, 18], Marco Gnesi[19], Mathias Holm[20], Christer Janson[21], Deborah Jarvis[6, 7], Ane Johannessen[22], Rudolf A. Jörres[1, 2], Stefan Karrasch[1, 2, 3], Benedicte Leynaert[23], José Antonio Maldonado Perez[24], Andrei Malinovschi[25], Jesús Martínez-Moratalla[26], Lars Modig[15], Dennis Nowak[1, 2], James Potts[6], Nicole Probst-Hensch[8, 9], José Luis Sánchez-Ramos[27], Valerie Siroux[11], Isabel Urrutia Landa[28], Danielle Vienneau[8, 9], Simona Villani[19], Bénédicte Jacquemin[29], Joachim Heinrich[1, 2, 30]
.

1. Institute and Clinic for Occupational, Social and Environmental Medicine, University Hospital, LMU Munich, Munich, Germany
2. Comprehensive Pneumology Center Munich (CPC-M), German Center for Lung Research (DZL), Munich, Germany
3. Institute of Epidemiology, Helmholtz Zentrum München - German Research Center for Environmental Health, Neuherberg, Germany
4. Institute of Psychology, Jagiellonian University, Krakow, Poland
5. “Health and quality of life in a green and sustainable environment”, SRIPD, Medical University of Plovdiv, Plovdiv, Bulgaria
6. National Heart and Lung Institute, Imperial College London, London, United Kingdom
7. MRC Centre for Environment & Health, London, UK
8. Swiss Tropical and Public Health Institute, Allschwil, Switzerland
9. University of Basel, Basel, Switzerland
10. Unit of Epidemiology and Medical Statistics, Department of Diagnostics and Public Health, University of Verona, Verona, Italy
11. University Grenoble Alpes, Inserm U 1209, CNRS UMR 5309, Team of Environmental Epidemiology Applied to the Development and Respiratory Health, Institute for Advanced Biosciences, Grenoble, France
12. Pediatric Department, CHU Grenoble Alpes, Grenoble, France
13. Social Epidemiology and Health Policy, Department of Family Medicine and Population Health, Faculty of Medicine and Health Sciences, University of Antwerp, Antwerp, Belgium
14. Institute for Environment and Sustainable Development (IMDO), University of Antwerp, Belgium
15. Section of Sustainable Health, Department of Public Health and Clinical Medicine, Umeå University, Umeå, Sweden
16. ISGlobal, Barcelona, Spain
17. Universitat Pompeu Fabra (UPF), Barcelona, Spain
18. CIBER Epidemiología y Salud Pública (CIBERESP), Barcelona, Spain
19. Unit of Biostatistics and Clinical Epidemiology, Department of Public Health, Experimental and Forensic Medicine, University of Pavia, Pavia, Italy
20. Occupational and Environmental Medicine, School of Public Health and Community Medicine, Institute of Medicine, Sahlgrenska Academy, University of Gothenburg, Gothenburg, Sweden
21. Department of Medical Sciences, Respiratory, Allergy and Sleep Research, Uppsala University, Uppsala, Sweden
22. Department of Global Public Health and Primary Care, University of Bergen, Bergen, Norway
23. Université Paris-Saclay, UVSQ, Univ. Paris-Sud, Inserm, Center for Epidemiology and Population Health (CESP) - Integrative Respiratory Epidemiology Team, 94807, Villejuif, France
24. Sección de Neumología, Hospital Juan Ramón Jiménez, Huelva, Spain
25. Department of Medical Sciences, Clinical Physiology, Uppsala University, Uppsala, Sweden
26. Servicio de Neumología del Complejo Hospitalario Universitario de Albacete, Albacete, Spain
27. Department of Nursing, University of Huelva, Huelva, Spain
28. Department of Pneumology, Galdakao Hospital, Galdakao, Spain
29. Univ Rennes, Inserm, EHESP, Irset (Institut de recherche en santé, environnement et travail), UMR_S 1085, F-35000 Rennes, France
30. School of Public Health and Preventive Medicine, Monash University, Melbourne, Australia

Contents

Figure S1. Flowchart of selection of study participants 3

Table S1. Spirometers used for lung function measurements at every ECRHS survey 4

eMethods: Covariates 5

Figure S2. Directed acyclic graph for variable selection 6

eMethods: Statistical modeling 7

Figure S3. Spearman correlation coefficients between exposures 8

Figure S5. Distribution of annual $PM_{2·5}$ concentrations across three ECRHS surveys and 17 centers........ 9

Figure S4. Distribution of annual NO_2 concentrations across three ECRHS surveys and 17 centers........ 10

Figure S6. Distribution of residential greenness (NDVI) in a 300m buffer across three ECRHS surveys and 17 centers. 11

Table S2. Additional associations between 7 µg/m³ increase in ambient ozone and lung function change 12

Local Principal Investigators, senior scientific teams and funding agencies for the European Community Respiratory Health Survey (ECRHS) 13

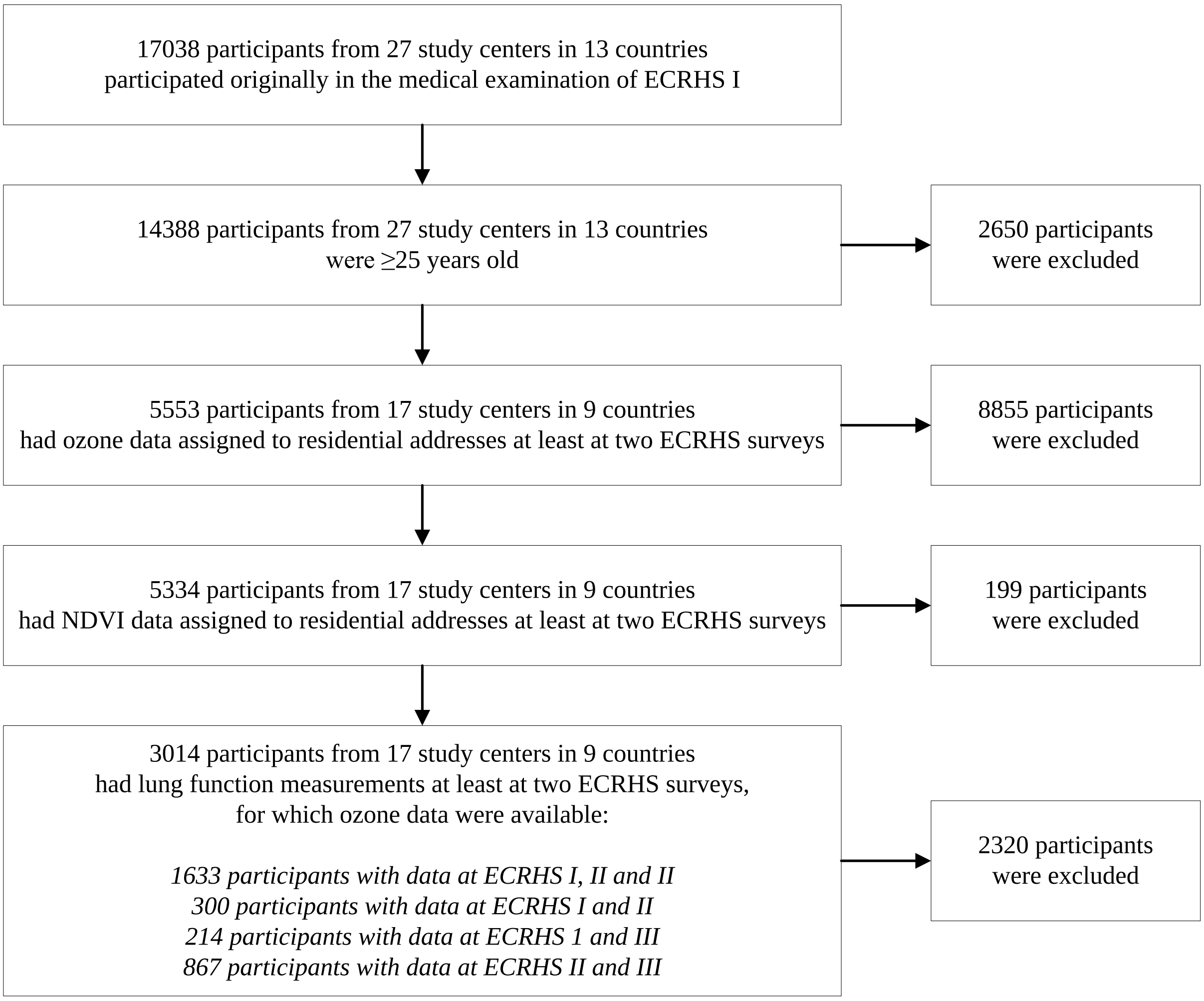

Figure S1. Flowchart of selection of study participants

Table S1. Spirometers used for lung function measurements at every ECRHS survey

Study center	ECRHS I	ECRHS II	ECRHS III
Umeå	SensorMedics displacement	SensorMedics displacement	NDD
Bergen	SensorMedics displacement	SensorMedics displacement	NDD
Uppsala	SensorMedics displacement	SensorMedics displacement	NDD
Gothenburg	SensorMedics displacement	SensorMedics displacement	NDD
Antwerp city	SensorMedics displacement	Jaeger pneumotach	NDD
South Antwerp	SensorMedics displacement	Jaeger pneumotach	NDD
Erfurt	Jaeger pneumotach	Jaeger pneumotach	NDD
Paris	Biomedin spiro	Biomedin spiro	NDD
Basel	SensorMedics hot wire	SensorMedics hot wire	NDD
Grenoble	Biomedin spiro	Biomedin spiro	NDD
Verona	Biomedin spiro	Biomedin spiro	Biomedin spiro
Pavia	Biomedin spiro	Biomedin spiro	NDD
Oviedo	Biomedin spiro	Biomedin spiro	NDD
Galdakao	Biomedin spiro	Biomedin spiro	NDD
Barcelona	Biomedin spiro	Biomedin spiro	NDD
Albacete	Biomedin spiro	Biomedin spiro	NDD
Huelva	Biomedin spiro	Biomedin spiro	NDD

eMethods: Covariates

We collected information on participants' characteristics, including type of sample (random, symptomatic), self-report current sex (female, male), age (years, at each survey), height (cm; at each survey), weight (kg, at each survey), and body mass index (BMI, kg/m^2, at each survey) and its categorization (normal, underweight: BMI < 25, overweight: 25 ≤ BMI ≤ 30, or obese: BMI >30, at each survey); season of study visit (warm: April to September, cold: October to March, at each survey); socioeconomic status measured by the age of completion of full-time education (the oldest age reported across ECRHS I and II: 17 years, 17-20 years, >20 years), and occupation (the highest occupational level reported across ECRHS II and III; management/professional/non-manual, technical/professional/non-manual, other non-manual, skilled manual, semi-skilled/unskilled manual, other/unknown); smoking status and lifetime pack-years (never smoker, ex-smoker < 15 pack-years, ex-smoker ≥ 15 pack-years, current smoker < 15 pack-years, current smoker ≥ 15 pack-years, at each survey); as well as urbanicity defined according to the standard EU Degree of Urbanisation[1] classification for the year 2001, which corresponds to the ECRHS II survey (urban/cities: densely populated areas, semi-urban/towns and suburbs: intermediate density areas, and rural areas: thinly populated areas, at each survey).

We also registered spirometer model (Table S1), baseline (ECRHS I) lung function, and geographic region (Umeå, Uppsala, Gothenburg, and Bergen as Northern Europe; Antwerp city, South Antwerp, Erfurt, Paris, Grenoble, Basel, Verona, and Pavia as Central Europe; and Oviedo, Galdakao, Barcelona, Albacete, and Huelva as Southern Europe).

Reference

1. Eeftens M, Beelen R, Fischer P, Brunekreef B, Meliefste K, Hoek G. Stability of measured and modelled spatial contrasts in NO(2) over time. *Occupational and environmental medicine* 2011; **68**(10): 765-70.

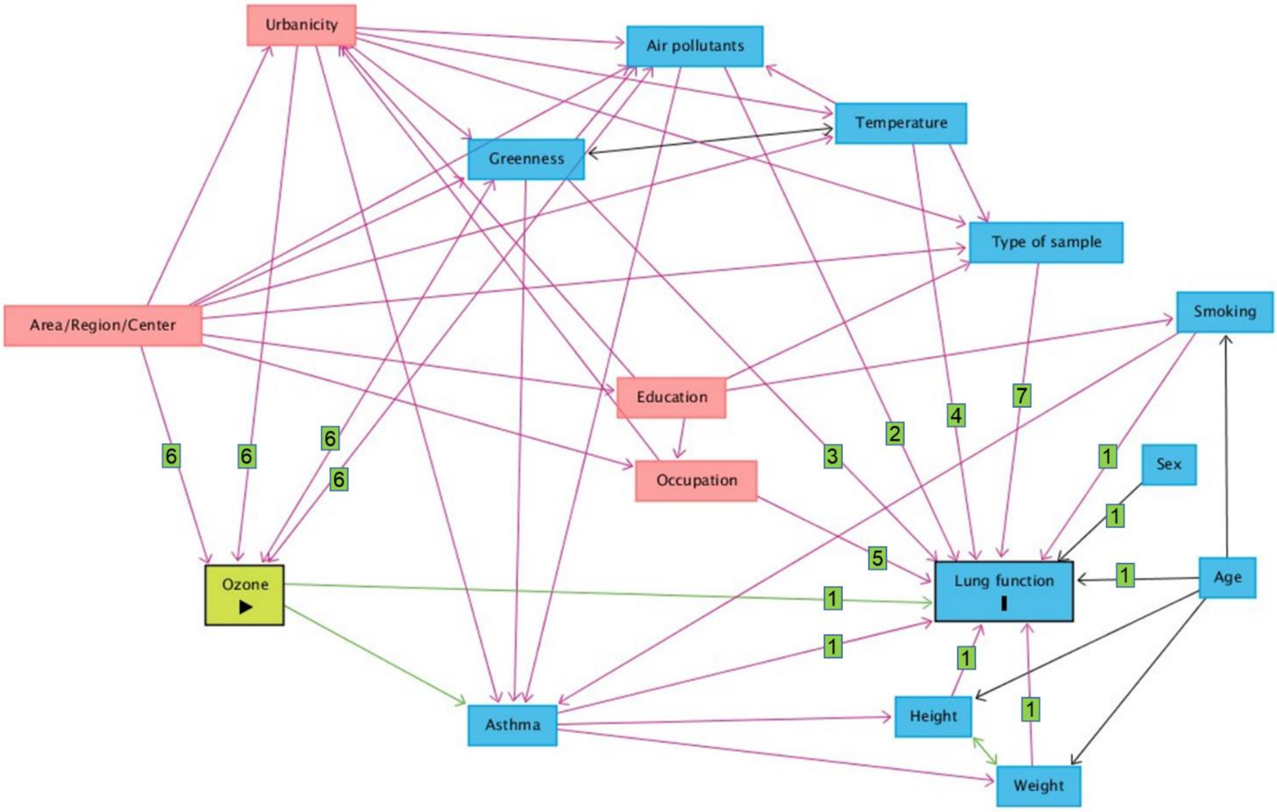

Figure S2. Directed acyclic graph for variable selection

The DAG is based on published literature and expert knowledge and consists of nodes and arrows representing variables and the causal associations between them, respectively. The backtracking algorithm enables the identification of a minimally sufficient adjustment set, which takes into account all important confounders needed for obtaining unbiased estimates.

The node labeled "Ozone" indicates the exposure, and the one with "Lung function" indicates the outcome. The green line pointing from the exposure to the outcome depicts the association of interest. The main associations derived from the literature are marked by a number in the DAG, and listed below:

1. Agusti A, Faner R. Lung function trajectories in health and disease. The Lancet Respiratory medicine 2019; 7(4): 358-64.
2. Adam M, Schikowski T, Carsin AE, et al. Adult lung function and long-term air pollution exposure. ESCAPE: a multicentre cohort study and meta-analysis. The European respiratory journal 2015; 45(1): 38-50.
3. Markevych I, Zhao T, Fuertes E, et al. Residential greenspace and lung function decline over 20 years in a prospective cohort: The ECRHS study. Environment international 2023; 178: 108036.
4. Rice MB, Li W, Wilker EH, et al. Association of outdoor temperature with lung function in a temperate climate. The European respiratory journal 2019; 53(1).
5. Ratanachina J, Amaral AFS, De Matteis S, et al. Association of respiratory symptoms and lung function with occupation in the multinational Burden of Obstructive Lung Disease (BOLD) study. The European respiratory journal 2023; 61(1).
6. Fitzky AC, Sandén H, Karl T, et al. The Interplay Between Ozone and Urban Vegetation—BVOC Emissions, Ozone Deposition, and Tree Ecophysiology. Frontiers in Forests and Global Change 2019; 2.
7. Janson C, Anto J, Burney P, et al. The European Community Respiratory Health Survey: what are the main results so far? European Community Respiratory Health Survey II. The European respiratory journal 2001; 18(3): 598-611.

eMethods: Statistical modeling

To capture the ozone-related change in spirometric parameters, our linear mixed effects regression models included an interaction term between long-term ozone exposure and age. We have used a three-level linear regression model: (1) level 1 = ECRHS survey (1st, 2nd or 3rd); (2) level 2 = subject; (3) level 3 = center, and with random intercept terms at levels 2 (subject) and 3 (center) and a 1st order autoregressive error at level 1:

$LuFu_{ijk} = \beta_0 + \beta_1 \times ECRHS_II_{ijk} + \beta_2 \times ECRHS_III_{ijk} + \beta_3 \times OZONE_{ijk} + \beta_4 \times AGE_{ijk} + \beta_5 \times AGE_{ijk}^2 + \boldsymbol{\beta_6 \times OZONE_{ijk} \times AGE_{ijk}} + \beta_7 \times OZONE_{ijk} \times AGE_{ijk}^2 + \ldots$other covariates$\ldots + z_{0i} + u_{0ij} + e_{0ijk}$

with k (ECRHS survey) = 1, 2, 3; j (subject)= 1, …, n_j; i (center) = 1, …, 17

Where:

$LuFu_{ijk}$ = Lung function measurement at ECRHS survey k for subject j from center i
$ECRHS\_II_{ijk}$ = dummy variable indicating 2nd ECRHS survey for subject j from center i
$ECRHS\_III_{ijk}$ = dummy variable indicating 3rd ECRHS survey for subject j from center i
$OZONE_{ijk}$ = ozone exposure at ECRHS survey k for subject j from center i
AGE_{ijk} = age at ECRHS survey k for subject j from centre i
AGE_{ijk}^2 = age-square at ECRHS survey k for subject j from center i
z_{0i} = level 3 residual
u_{0ij} = level 2 residual
e_{0ijk} = level 1 residual

Specifically, by including the interaction term between ozone exposure and age ($\beta_6 \times OZONE_{ijk} \times AGE_{ijk}$), the model accounts for the potential effect modification of age on the association between ozone exposure and lung function change.

	Ozone_ECRHS I	Ozone_ECRHS II	Ozone_ECRHS II	$PM_{2.5}$_ECRHS I	$PM_{2.5}$_ECRHS II	$PM_{2.5}$_ECRHS III	NO_2_ECRHS I	NO_2_ECRHS II	NO_2_ECRHS III	NDVI-300_ECRHS I	NDVI-300_ECRHS II	NDVI-300_ECRHS III
Ozone_ECRHS I	1.00											
Ozone_ECRHS II	0.77	1.00										
Ozone_ECRHS II	0.83	0.91	1.00									
$PM_{2.5}$_ECRHS I	-0.19	-0.09	-0.11	1.00								
$PM_{2.5}$_ECRHS II	-0.14	-0.15	-0.14	0.96	1.00							
$PM_{2.5}$_ECRHS III	-0.16	-0.13	-0.16	0.97	0.98	1.00						
NO_2_ECRHS I	-0.43	-0.23	-0.27	0.89	0.85	0.86	1.00					
NO_2_ECRHS II	-0.32	-0.44	-0.39	0.83	0.88	0.86	0.83	1.00				
NO_2_ECRHS III	-0.35	-0.37	-0.45	0.84	0.86	0.88	0.87	0.93	1.00			
NDVI-300_ECRHS I	0.28	0.14	0.18	-0.55	-0.51	-0.52	-0.65	-0.52	-0.55	1.00		
NDVI-300_ECRHS II	0.23	0.25	0.31	-0.56	-0.59	-0.60	-0.61	-0.66	-0.71	0.61	1.00	
NDVI-300_ECRHS III	0.27	0.41	0.34	-0.46	-0.52	-0.49	-0.48	-0.65	-0.56	0.48	0.69	1.00

1.00	0.80	0.60	0.40	0.20	0.00	-0.20	-0.40	-0.60	-0.80	-1.00

Figure S3. Spearman correlation coefficients between exposures

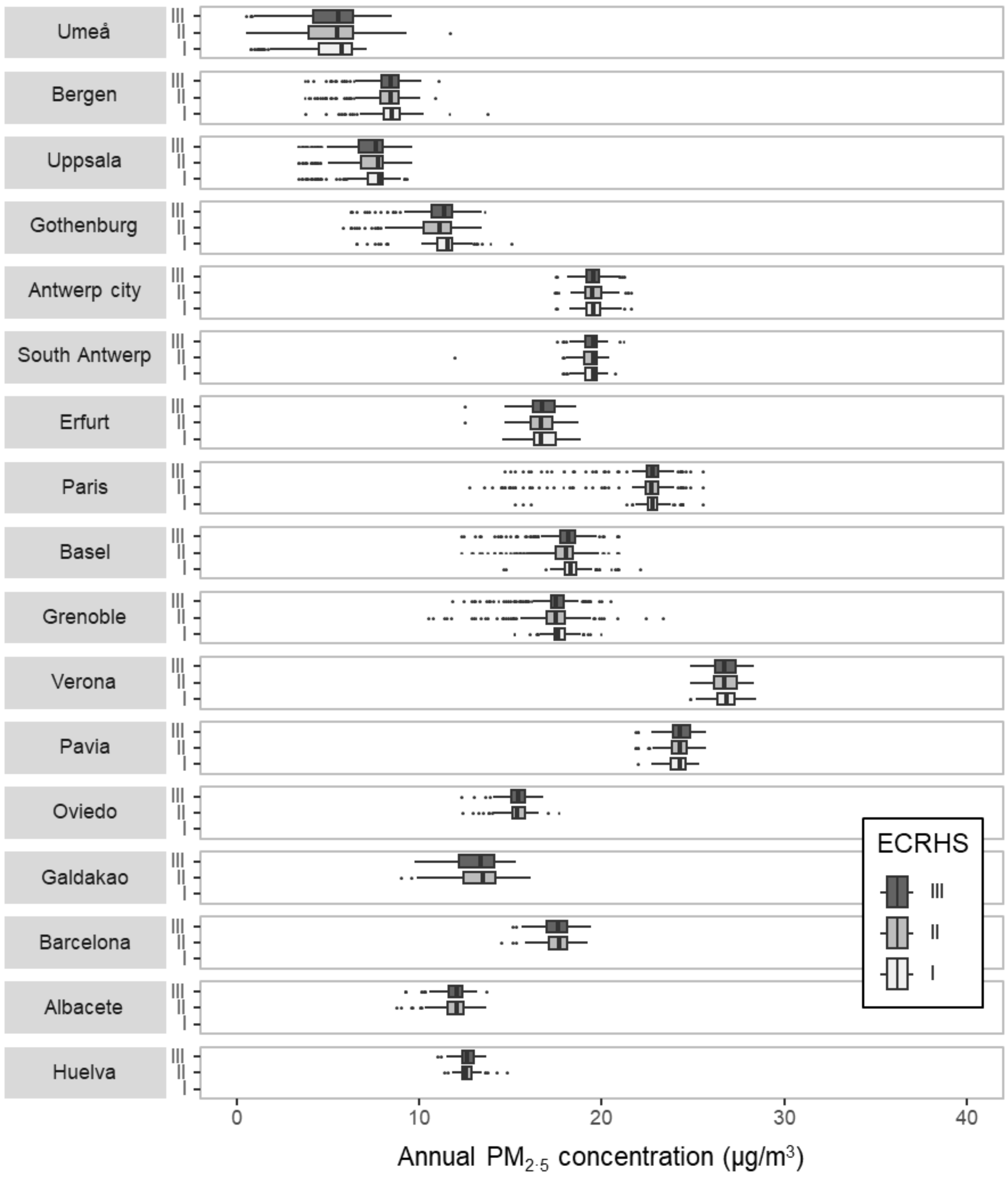

Figure S5. Distribution of annual PM2·5 concentrations across three ECRHS surveys and 17 centers. ECRHS centers are ranked from the north (top) to the south (bottom). For each boxplot, the black line indicates the median value, and the box summarizes the 25th to 75th percentiles.

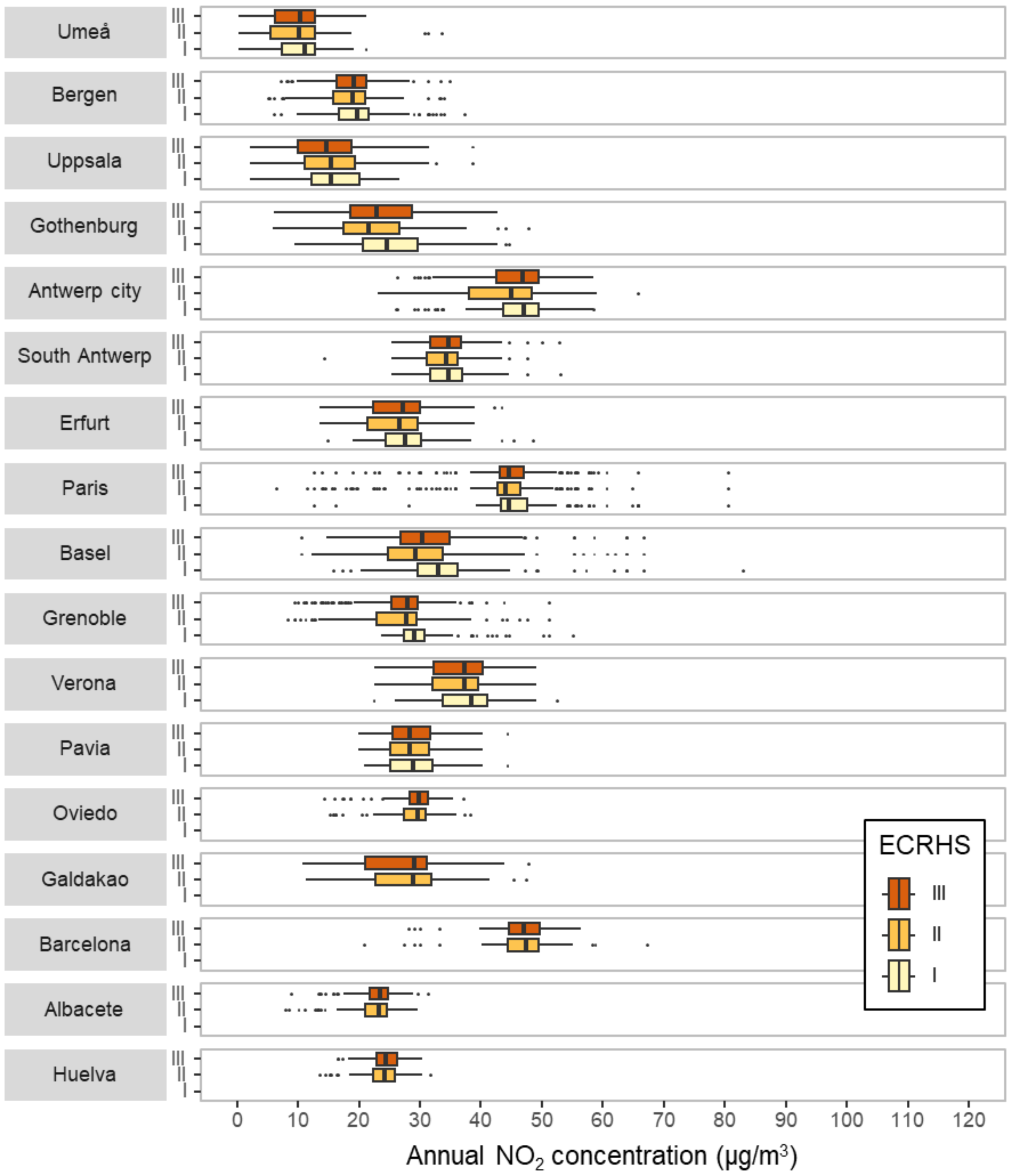

Figure S4. Distribution of annual NO_2 concentrations across three ECRHS surveys and 17 centers. ECRHS centers are ranked from the north (top) to the south (bottom). For each boxplot, the black line indicates the median value, and the box summarizes the 25th to 75th percentiles.

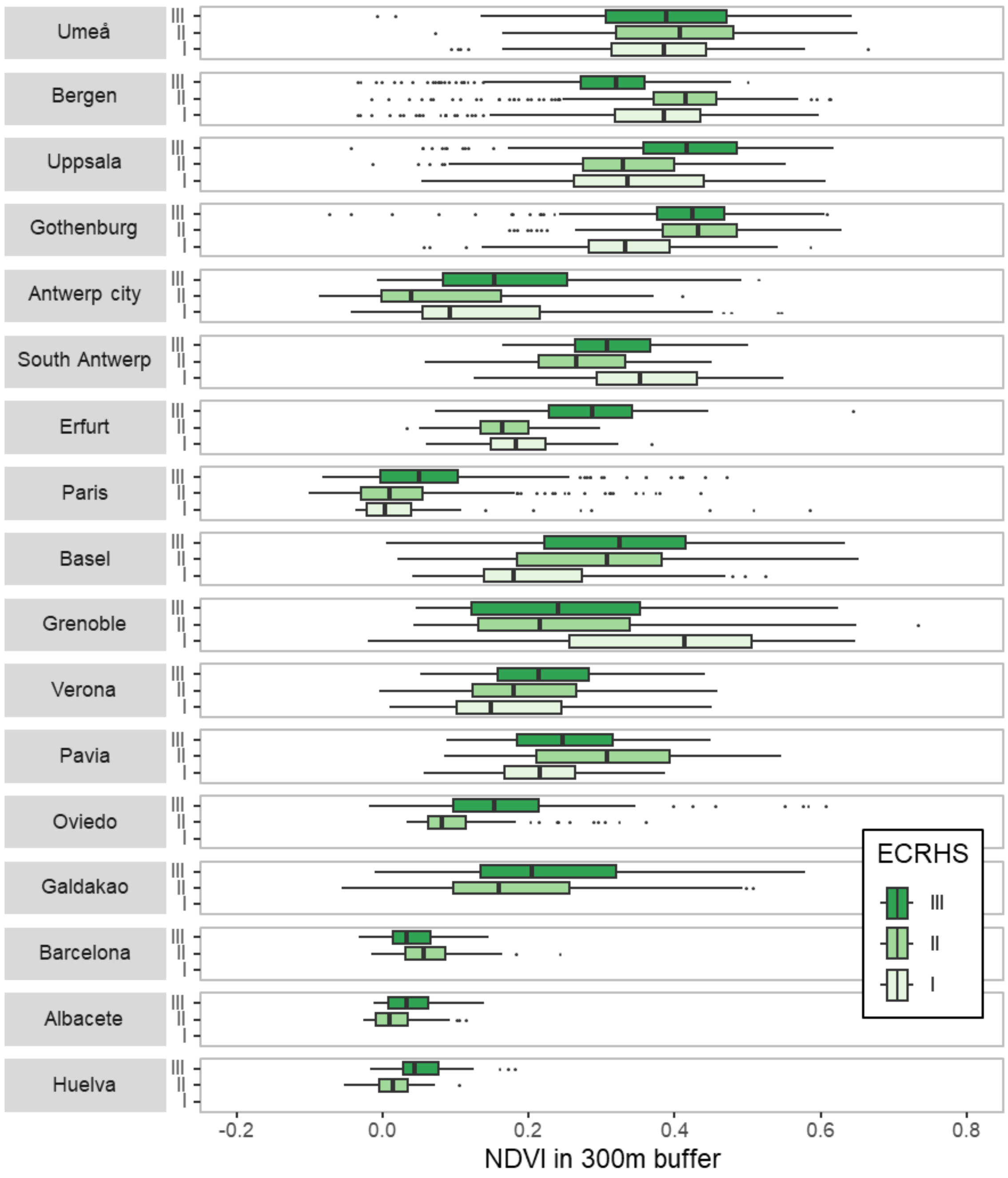

Figure S6. Distribution of residential greenness (NDVI) in a 300m buffer across three ECRHS surveys and 17 centers.

ECRHS centers are ranked from the north (top) to the south (bottom). For each boxplot, the black line indicates the median value, and the box summarizes the 25th to 75th percentiles.

Table S2. Additional associations between 7 μg/m³ increase in ambient ozone and lung function change

Model[a]	FEV$_1$ (mL/year)			FVC (mL/year)			FEV$_1$/FVC (%/year)		
	**n**	**beta**	**95% CI**	**n**	**beta**	**95% CI**	**n**	**beta**	**95% CI**
Main + Season[b]	**2909**	**-2·01**	**(-2·73, -1·30)**	**2909**	**-2·77**	**(-3·63, -1·90)**	2909	0·005	(-0·007, 0·017)
Main with continuous BMI[c]	**2909**	**-2·05**	**(-2·76, -1·33)**	**2909**	**-2·81**	**(-3·69, -1·94)**	2909	0·005	(-0·007, 0·017)
Main with categorized BMI[d]	**2909**	**-2·06**	**(-2·79, -1·34)**	**2909**	**-2·85**	**(-3·74, -1·96)**	2909	0·005	(-0·007, 0·018)
Random sample	**2499**	**-1·97**	**(-2·73·, -1·22)**	**2499**	**-2·79**	**(-3·71, -1·88)**	2499	0·011	(-0·001, 0·024)
Without Spanish centers	**2304**	**-1·80**	**(-2·68, -0·91)**	**2304**	**-3·47**	**(-4·55, -2·40)**	**2304**	**0·029**	**(0·014, 0·044)**
Main, fixed effect of center[e]	**2909**	**-2·07**	**(-2·79, -1·35)**	**2909**	**-2·86**	**(-3·73, -1·20)**	2909	0·005	(-0·007, 0·018)

Abbreviations: CI, confidence interval; FEV$_1$, forced expiratory volume in 1 second; FVC, forced vital capacity; NDVI, Normalized Difference Vegetation Index; NO_2, nitrogen dioxide; $PM_{2·5}$, particulate matter with aerodynamic diameter < 2·5 μm.

Boldface indicates p value < 0·05.

[a] Sample size of different models changes due to data availability; associations were assess by mixed linear models with an interaction term between long-term ozone exposure and age.

[b] With random intercepts for subjects nested within centers, and adjusted for sample, sex, age, age squared, height, weight, age at completion of full-time education, occupation, and smoking status and lifetime pack-years smoked, as well as season.

[c] With random intercepts for subjects nested within centers, and adjusted for sample, sex, age, age squared, height, weight, age at completion of full-time education, occupation, and smoking status and lifetime pack-years smoked, as well as BMI.

[d] With random intercepts for subjects nested within centers, and adjusted for sample, sex, age, age squared, height, weight, age at completion of full-time education, occupation, and smoking status and lifetime pack-years smoked, as well as BMI categorization.

[e] With random intercepts for subjects and fixed effect of center, and adjusted for sample, sex, age, age squared, height, weight, age at completion of full-time education, occupation, and smoking status and lifetime pack-years smoked.

Local Principal Investigators, senior scientific teams and funding agencies for the European Community Respiratory Health Survey (ECRHS)

ECRHS

Co-ordinating center: P. Burney, S. Chinn, C. Luczynska, D. Jarvis, E. Lai (London).

Project management group: P. Burney (Project leader-UK), S. Chinn (UK), C. Luczynska† (UK), D. Jarvis (UK), P. Vermeire (Antwerp), H. Kesteloot (Leuven), J. Bousquet (Montpellier), D. Nowak (Hamburg), J. Prichard (Dublin), R. de Marco (Verona), B. Rijcken (Groningen), J.M. Anto (Barcelona), J. Alves (Oporto), G. Boman (Uppsala), N. Nielsen (Copenhagen), P. Paoletti (Pisa).

Steering Committee of ECRHS II: U. Ackermann-Liebrich (University of Basel, Switzerland); N. Kuenzli (University of Basel, and University of Southern California, Los Angeles, USA); J.M. Antó and J. Sunyer (Institut Municipal d'Investigació Médica (IMIM-IMAS), Universitat Pompeu Fabra (UPF), Spain); P. Burney (project leader), S Chinn, D. Jarvis, J. Knox and C. Luczynska† (King's College London, UK); I. Cerveri (University of Pavia, Italy); R. de Marco† (University of Verona, Italy); T. Gislason (Iceland University Hospital, Iceland); J. Heinrich and M. Wjst (GSF–Institute of Epidemiology, Germany); C. Janson (Uppsala University, Sweden); B. Leynaert and F. Neukirch (Institut National de la Sante´ et de la Recherche Medicale (INSERM), France); J. Schouten (University of Groningen, The Netherlands); C. Svanes (University of Bergen, Norway); P. Vermeire† (University of Antwerp, Belgium).

Principal investigators and senior scientific teams of ECRHS II: Australia: (M. Abramson, E.H Walters, J. Raven); Belgium: South Antwerp and Antwerp City (P. Vermeire†, J. Weyler, M. van Sprundel, V. Nelen); Estonia: Tartu (R. Jõgi, A. Soon); France: Paris (F. Neukirch, B. Leynaert, R. Liard, M. Zureik), Grenoble (I. Pin, J. Ferran-Quentin); Bordeaux (A. Taytard, C. Raherison), Montpellier (J. bBousquet, P.J. Bousquet); Germany: Erfurt (J. Heinrich, M. Wjst, C. Frye, I. Meyer); Iceland: Reykjavik (T. Gislason, E. Bjornsson, D. Gislason, K.B. Jörundsdóttir); Italy: Turin (R. Bono, M. Bugiani, P.Piccioni, E. Caria, A. Carosso, E. Migliore, G. Castiglioni), Verona (R. de Marco†, G. Verlato, E. Zanolin, S. Accordini, A. Poli, V. Lo Cascio, M. Ferrari, I. Cazzoletti), Pavia (A. Marinoni, S. Villani, M. Ponzio, F. Frigerio, M. Comelli, M. Grassi, I. Cerveri, A. Corsico); The Netherlands: Groningen and Geleen (J. Schouten, M. Kerkhof); Norway: Bergen (A. Gulsvik, E. Omenaas, C. Svanes, B. Laerum); Spain: Albacete (J. Martinez-Moratalla Rovira, E. Almar, M. Arévalo, C. Boix, G. González, J.M. Ignacio García, J. Solera, J. Damián), Galdakao (N. Muñozguren, J. Ramos, I. Urrutia, U. Aguirre), Barcelona (J.M. Antó, J. Sunyer, M. Kogevinas, J.P. Zock, X. Basagaña, A. Jaen, F. Burgos, C. Acosta), Huelva (J. Maldonado, A. Pereira, J.L. Sanchez), Oviedo (F. Payo, I. Huerta, A. de la Vega, L. Palenciano, J. Azofra, A. Cañada); Sweden: Göteborg (K. Toren, L. Lillienberg, A.C. Olin, B. Balder, A. Pfeifer-Nilsson, R. Sundberg), Umeå (E. Norrman, M. Soderberg, K.A. Franklin, B. Lundback, B. Forsberg, L. Nystrom), Uppsala (C. Janson, G. Boman, D. Norback, G. Wieslander, M. Gunnbjornsdottir); Switzerland: Basel (N. Küenzli, B. Dibbert, M. Hazenkamp, M. Brutsche, U. Ackermann-Liebrich); United Kingdom: Ipswich (D. Jarvis, R. Hall, D. Seaton), Norwich (D. Jarvis, B. Harrison).

Principal Investigators and senior scientific teams of ECRHS III: Australia: Melbourne (M. Abramson, G. Benke, S. Dharmage, B. Thompson, S. Kaushik, M. Matheson); Belgium: South Antwerp & Antwerp City (J. Weyler, H. Bentouhami, V. Nelen); Denmark: Aarhus (V. Schlunssen, T. Sigsgaard, R.Dahl); Estonia: Tartu (R. Jõgi, H.Orru); France: Bordeaux (C. Raherison, P.O. Girodet), Grenoble (I. Pin, V. Siroux, J. Ferran, J.L. Cracowski), Montpellier (P. Demoly, A.Bourdin, I. Vachier), Paris (B. Leynaert, D. Soussan, D. Courbon, C. Neukirch, L. Alavoine, X. Duval, I. Poirier); Germany: Erfurt (J. Heinrich, E. Becker, G. Woelke, O. Manuwald), Hamburg (H. Magnussen, D. Nowak, A.M. Kirsten); Iceland: Reykjavik (T. Gislason, B. Benediktsdottir, D. Gislason, E.S. Arnardottir, M. Clausen, G. Gudmundsson, L. Gudmundsdottir, H. Palsdottir, K. Olafsdottir, S. Sigmundsdottir, K. Bara-Jörundsdottir); Italy: Pavia (I. Cerveri, A.Corsico, A. Grosso, F. Albicini, E. Gini, E.M. Di Vincenzo, V. Ronzoni, S. Villani, F. Campanella, M. Gnesi, F. Manzoni, L. Rossi, O. Ferraro),Turin: (M. Bugiani, R. Bono, P. Piccioni, R. Tassinari, V. Bellisario, G. Trucco), Verona: (R de Marco†, S. Accordini, L. Calciano, L. Cazzoletti, M. Ferrari, A.M. Fratta Pasini, F. Locatelli, P. Marchetti, A. Marcon, E. Montoli, G. Nguyen, M. Olivieri, C. Papadopoulou, C.Posenato, G. Pesce, P. Vallerio, G. Verlato, E. Zanolin); Netherlands: Groningen (H.M. Boezen); Norway: (C. Svanes, E. Omenaas, A. Johannessen, T. Skorge, F. Gomez Real); Spain: Albacete (J. Martinez-Moratalla Rovira, E. Almar, A. Mateos, S. García, A. Núñez, P. López, R. Sánchez, E. Mancebo), Barcelona:(J.M. Antó, J.P. Zock, J. Garcia-Aymerich, M. Kogevinas, X. Basagaña, A.E. Carsin, F. Burgos, C. Sanjuas, S. Guerra, B. Jacquemin, P. Davdand); Galdakao (N. Muñozguren, I. Urrutia, U. Aguirre, S. Pascual); Huelva (J. Antonio Maldonado, A. Pereira, J. Luis Sánchez, L. Palacios); Oviedo (F. Payo, I. Huerta, N. Sánchez, M. Fernández, B. Robles); Sweden: Göteborg (K. Torén, M. Holm, J.L. Kim, A.C. Olin, A. Dahlman-Höglund), Umeå (B. Forsberg, L. Braback, L. Modig, B. Järvholm, H. Bertilsson, K.A. Franklin, C. Wahlgreen), Uppsala:(B. Andersson, D. Norback, U. Spetz

Nystrom, G. Wieslander, G.M. Bodinaa Lund, K Nisser); Switzerland: Basel (N.M. Probst-Hensch, N. Künzli, D. Stolz, C. Schindler, T. Rochat, J.M. Gaspoz, E. Zemp Stutz, M. Adam, C. Autenrieth, I. Curjuric, J. Dratva, A. Di Pasquale, R. Ducret-Stich, E. Fischer, L. Grize, A. Hensel, D. Keidel, A. Kumar, M. Imboden, N. Maire, A. Mehta, H. Phuleria, M. Ragettli, M. Ritter, E. Schaffner, G.A. Thun, A. Ineichen, T. Schikowski, M. Tarantino, M. Tsai); UK: London (P. Burney, D. Jarvis, S. Kapur, R. Newson, J. Potts), Ipswich (N. Innes), Norwich (A. Wilson).

Financial support for ECRHS I: The co-ordination of this work was supported by the European Commission and the authors and participants are grateful to the late C. Baya and M. Hallen for their help during the study and K. Vuylsteek and the members of the COMAC for their support. The following grants helped to fund the local studies: Australia: Asthma Foundation of Victoria, Allen+Hanburys; Belgium: Belgian Science Policy Office, National Fund for Scientific Research; Estonia: Estonian Science Foundation, grant no 1088; France: Ministère de la Santé, Glaxo France, Institut Pneumologique d'Aquitaine, Contrat de Plan Etat-Région Languedoc-Rousillon, CNMATS, CNMRT (90MR/10, 91AF/6), Ministre delegué de la santé, RNSP, France, GSF; Germany: Bundesminister für Forschung und Technologie; Italy: Ministero dell'Università e della Ricerca Scientifica e Tecnologica, CNR, Regione Veneto grant RSF n. 381/05.93; Norway: Norwegian Research Council project no. 101422/310; Portugal: Glaxo Farmacêutica Lda, Sandoz Portugesa; Spain: Fondo de Investigación Sanitaria (#91/0016-060-05/E, 92/0319 and #93/0393), Hospital General de Albacete, Hospital General Juan Ramón Jiménez, Dirección Regional de Salud Pública (Consejería de Sanidad del Principado de Asturias), CIRIT (1997 SGR 00079) and Servicio Andaluz de Salud; Sweden: The Swedish Medical Research Council, the Swedish Heart Lung Foundation, the Swedish Association against Asthma and Allergy; Switzerland: Swiss National Science Foundation grant 4026-28099; UK: National Asthma Campaign, British Lung Foundation, Department of Health, South Thames Regional Health Authority.

Financial Support for ECRHS II: Australia: National Health & Medical Research Council; Belgium: Antwerp: Fund for Scientific Research (grant code, G.0402.00), University of Antwerp, Flemish Health Ministry; Estonia: Tartu Estonian Science Foundation grant no 4350, France: (All) Programme Hospitalier de Recherche Clinique—Direction de la Recherche Clinique (DRC) de Grenoble 2000 number 2610, Ministry of Health, Ministère de l'Emploi et de la Solidarité, Direction Génerale de la Santé, Centre Hospitalier Universitaire (CHU) de Grenoble; Bordeaux: Institut Pneumologique d'Aquitaine; Grenoble: Comite des Maladies Respiratoires de l'Isere Montpellier: Aventis (France), Direction Regionale des Affaires Sanitaires et Sociales Languedoc-Roussillon; Paris: Union Chimique Belge-Pharma (France), Aventis (France), Glaxo France, Germany: Erfurt: GSF—National Research Centre for Environment and Health, Deutsche Forschungsgemeinschaft (grant code, FR1526/1-1); Hamburg: GSF—National Research Centre for Environment and Health, Deutsche Forschungsgemeinschaft (grant code, MA 711/4-1); Iceland: Reykjavik, Icelandic Research Council, Icelandic University Hospital Fund; Italy: Pavia: GlaxoSmithKline Italy, Italian Ministry of University and Scientific and Technological Research (MURST), Local University Funding for Research 1998 and 1999; Turin: Azienda Sanitaria Locale 4 Regione Piemonte (Italy), Azienda Ospedaliera Centro Traumatologico Ospedaliero/Centro Traumatologico Ortopedico—Istituto Clinico Ortopedico Regina Maria Adelaide Regione Piemonte; Verona: Ministero dell'Università e della Ricerca Scientifica (MURST), Glaxo Wellcome s.p.a.; Norway: Bergen: Norwegian Research Council, Norwegian Asthma and Allergy Association, Glaxo Wellcome AS, Norway Research Fund; Spain: Fondo de Investigacion Santarias (grant codes, 97/0035-01, 99/0034-01 and 99/0034-02), Hospital Universitario de Albacete, Consejeria de Sanidad; Barcelona: Sociedad Espanola de Neumología y Cirugía Toracica, Public Health Service (grant code, R01 HL62633-01), Fondo de Investigaciones Santarias (grant codes, 97/0035-01, 99/0034-01, and 99/0034-02), Consell Interdepartamentalde Recerca i Innovació Tecnológica (grant code, 1999SGR 00241) Instituto de Salud Carlos III; Red de Centros de Epidemiología y Salud Pública, C03/09,Red de Bases moleculares y fisiológicas de las Enfermedades Respiratorias, C03/011and Red de Grupos Infancia y Medio Ambiente G03/176; Huelva: Fondo de Investigaciones Santarias (grant codes, 97/0035-01, 99/0034-01, and 99/0034-02); Galdakao: Basque Health Department; Oviedo: Fondo de Investigaciones Sanitaria (97/0035-02, 97/0035, 99/0034-01, 99/0034-02, 99/0034-04, 99/0034-06, 99/350, 99/0034--07), European Commission (EU-PEAL PL01237), Generalitat de Catalunya (CIRIT 1999 SGR 00214), Hospital Universitario de Albacete, Sociedad Española de Neumología y Cirugía Torácica (SEPAR R01 HL62633-01) Red de Centros de Epidemiología y Salud Pública (C03/09), Red de Bases moleculares y fisiológicas de las Enfermedades Respiratorias (C03/011) and Red de Grupos Infancia y Medio Ambiente (G03/176; 97/0035-01, 99/0034-01, and 99/0034-02); Sweden: Göteborg, Umeå, Uppsala: Swedish Heart Lung Foundation, Swedish Foundation for Health Care Sciences and Allergy Research, Swedish Asthma and Allergy Foundation, Swedish Cancer and Allergy Foundation, Swedish Council for Working Life and Social Research (FAS); Switzerland: Basel Swiss National Science Foundation, Swiss Federal Office for Education and Science, Swiss National Accident Insurance Fund; UK: Ipswich and Norwich: Asthma UK (formerly known as National Asthma Campaign).

Financial Support for ECRHS III: Australia: National Health & Medical Research Council; Belgium: Antwerp South and Antwerp City: Research Foundation Flanders (FWO), grant code G.0.410.08.N.10; Estonia: Tartu: SF0180060s09 from the Estonian Ministry of Education; France: (All) Ministère de la Santé, Programme Hospitalier de Recherche Clinique (PHRC) national 2010, Bordeaux: INSERM U897 Université Bordeaux segalen,

Grenoble: Comite Scientifique AGIRadom 2011, Paris: Agence Nationale de la Santé, Région Ile de France, domaine d'intérêt majeur (DIM); Germany: Erfurt: German Research Foundation HE 3294/10-1, Hamburg: German Research Foundation MA 711/6-1, NO 262/7-1; Iceland: Reykjavik: The Landspitali University Hospital Research Fund, University of Iceland Research Fund, ResMed Foundation, California, USA, Orkuveita Reykjavikur (Geothermal plant), Vegagerðin (The Icelandic Road Administration (ICERA); Italy: All Italian centres were funded by the Italian Ministry of Health, Chiesi Farmaceutici SpA, in addition Verona was funded by Cariverona foundation, Education Ministry (MIUR); Norway: Norwegian Research council grant no 214123, Western Norway Regional Health Authorities grant no 911631, Bergen Medical Research Foundation; Spain: Fondo de Investigación Sanitaria (PS09/02457, PS09/00716, PS09/01511, PS09/02185, PS09/03190), Servicio Andaluz de Salud, Sociedad Española de Neumología y Cirurgía Torácica (SEPAR 1001/2010), Fondo de Investigación Sanitaria (PS09/02457), Barcelona: Fondo de Investigación Sanitaria (FIS PS09/00716), Galdakao: Fondo de Investigación Sanitaria (FIS 09/01511), Huelva: Fondo de Investigación Sanitaria (FIS PS09/02185) and Servicio Andaluz de Salud, Oviedo: Fondo de Investigación Sanitaria (FIS PS09/03190); Sweden: All centres were funded by The Swedish Heart and Lung Foundation, The Swedish Asthma and Allergy Association, The Swedish Association against Lung and Heart Disease, Swedish Research Council for health, working life and welfare (FORTE), Göteborg: also received further funding from the Swedish Council for Working life and Social Research. Umeå: also received funding from Vasterbotten Country Council ALF grant; Switzerland: The Swiss National Science Foundation (grants no 33CSCO-134276/1, 33CSCO-108796, 3247BO-104283, 3247BO-104288, 3247BO-104284, 3247-065896, 3100-059302, 3200-052720, 3200-042532, 4026-028099), ECRHS was funded in the context of the Swiss National Science Foundation Grant 33CS30_177506, The Federal office for forest, environment and landscape, The Federal Office of Public Health, The Federal Office of Roads and Transport, the canton's government of Aargan, Basel-Stadt, Basel-Land, Geneva, Luzern, Ticino, Valais and Zürich, the Swiss Lung League, the canton's Lung League of Basel Stadt/ Basel, Landschaft, Geneva, Ticino, Valais and Zurich, SUVA, Freiwillige Akademische Gesellschaft, UBS Wealth Foundation, Talecris Biotherapeutics GmbH, Abbott Diagnostics, European Commission 018996 (GABRIEL), Wellcome Trust WT 084703MA; UK: Medical Research Council (Grant Number 92091), support also provided by the National Institute for Health Research through the Primary Care Research Network.
